# Healthcare and health situation of adults with type 2 diabetes in Germany: The study GEDA 2021/2022-Diabetes

**DOI:** 10.25646/12128

**Published:** 2024-06-19

**Authors:** Christin Heidemann, Yong Du, Elvira Mauz, Lena Walther, Diana Peitz, Anja Müller, Maike Buchmann, Jennifer Allen, Christa Scheidt-Nave, Jens Baumert

**Affiliations:** Robert Koch Institute, Department of Epidemiology and Health Monitoring, Berlin, Germany

**Keywords:** Diabetes mellitus, Surveillance, Treatment, Psyche, Health, COVID-19, Germany

## Abstract

**Background:**

The nationwide study German Health Update (GEDA) 2021/2022-Diabetes was conducted to assess the current healthcare and health situation of adults with diabetes in Germany.

**Methods:**

GEDA 2021/2022-Diabetes comprises a sample of adults with diagnosed diabetes from the general population. The analysis focuses on adults aged 45 years and over with type 2 diabetes (N = 1,448) and provides selected indicators on diabetes care as well as mental, social and general health.

**Results:**

87.5 % of participants aged 45 years and over with type 2 diabetes are treated with blood glucose-lowering medication. 36.5 % receive insulin alone or in combination with other antidiabetics; 0.7 % use an insulin pump. Almost 96 % had an HbA1c measurement in the last year and about two thirds each report annual foot and eye examinations, participation in a diabetes self-management education programme and self-monitoring of their feet and of blood glucose (12.0 % with continuous glucose monitoring). On average, the quality of diabetes care is perceived as moderate. 23.8 % rate their mental health as excellent/very good. More than a tenth each have anxiety or depressive symptoms and feelings of loneliness. Half rate their general health as very good/good.

**Conclusions:**

There is a potential for improvement in the quality of diabetes care and the mental and physical health of adults with type 2 diabetes.

## 1. Introduction

In Germany, about seven million people live with diabetes mellitus, which is a chronic metabolic disease characterized by elevated blood sugar levels [1]. Every year, around 500,000 people are newly diagnosed with diabetes [2, 3]. Over 90 % of people with diabetes have a type 2 diabetes, which usually develops in middle or older adulthood [2, 4]. The high public health relevance of type 2 diabetes is related, on the one hand, to these high case numbers, which according to forecasts will continue to rise due to demographic ageing in Germany alone [5], and, on the other hand, to the potential preventability of important behavioural risk factors of type 2 diabetes (e.g. unfavorable dietary and activity behaviour and associated overweight) as well as some adverse environmental and social conditions (e.g. air pollution and social deprivation) [6].

To improve the regulation of blood glucose levels in people with type 2 diabetes, depending on the risk profile and individual therapy goals, non-pharmacological treatment is achieved through changes in dietary or activity behaviour and, if this alone is not sufficient, treatment with blood glucose-lowering medication (antidiabetics) is prescribed. For pharmagological treatment, tablets (oral antidiabetics) or medications to be administered into the subcutaneous fatty tissue (mainly injectable GLP-1 receptor agonists) are used. In progressed type 2 diabetes, i.e. when the effect of the vital, blood sugar-regulating hormone insulin is reduced (insulin resistance) in conjunction with a substantial reduction in the body’s own insulin secretion, treatment is performed with insulin preparations to be administered into the subcutaneous fatty tissue alone or in combination with other antidiabetics [7]. An extended classification of type 2 diabetes into different subtypes is currently being discussed, which could lead to a more targeted therapy [8]. Diabetes that remains undetected for a long period of time or is inadequately controlled can lead to serious complications. Acute metabolic disturbances can occur, i.e. too low blood sugar levels (hypoglycemia) or too high blood sugar levels (hyperglycemia or ketoacidosis) and even impairment of consciousness and unconsciousness (diabetic coma) [9, 10]. In the long term, various diabetes-related secondary diseases can develop, in particular eye diseases up to blindness, kidney diseases up to kidney failure as well as nerve diseases and circulatory disorders up to amputations [11]. In addition, concomitant diseases (comorbidities) can be present, including cardiovascular diseases and psychological impairments, such as depressive conditions and anxiety disorders [11–13]. Diabetes and its secondary and concomitant diseases can be associated with a reduced quality of life and lower life expectancy [11, 14, 15].

Therefore, continuous diabetes treatment that is adapted to individual needs and active self-management by those affected is necessary in order to achieve optimal quality of care and avoid or delay the consequences of the disease [7]. Furthermore, the social integration of those affected and support from their personal environment can be important resources in dealing with the disease and for their general and mental well-being. For example, a higher level of social support is not only associated with a lower risk of type 2 diabetes [16], but also with more favorable clinical parameters (HbA1c, lipids, blood pressure), better self-management of the disease and more diet-conscious and active behaviour in people with diabetes [17]. Loneliness as a perceived absence of involvement in the social environment is associated with an increased risk of developing type 2 diabetes [18] and is also considered a risk factor for cardiovascular disease in people with diabetes [19].

Against this background, the nationwide survey German Health Update (GEDA) 2021/2022-Diabetes was conducted as part of the diabetes surveillance at the Robert Koch Institute (RKI) [20] and provides comprehensive information on the healthcare and health situation of adults with diabetes based on self-reported data. The analysis performed on this database aims to provide an up-to-date overview of selected key measures (indicators) regarding treatment, healthcare, self-assessed quality of care, mental health, social integration and self-assessed general health of people aged 45 years and over with type 2 diabetes in Germany.

As the survey was conducted at the turn of 2021/2022, the results provide an insight into the health situation of adults with diabetes in Germany at a time when the epidemic COVID-19 situation ‘of national scope’ had just been announced to be ended [21], however, due to the predominant Omicron variant, there were high 7-day incidences [22, 23] and therefore some infection control measures still in place [21]. As previous analyses indicate that adults in Germany with a history of SARS-CoV-2 infection rate their health worse than people without infection [24], the self-assessed change in health compared to the time before the pandemic was also considered, differentiated according to the reported SARS-CoV-2 infection status.

## 2. Method

### 2.1 Study design and sample

The GEDA 2021/2022-Diabetes study (Infobox) is based on a nationwide telephone sample in which randomly selected German-speaking people aged 18 years and over from the general population with self-reported, physician-diagnosed diabetes were interviewed. The cross-sectional survey was conducted from December 7, 2021 to April 9, 2022 via a telephone interview using a programmed, fully structured questionnaire (Computer Assisted Telephone Interview, CATI). The sampling and data collection were carried out on behalf of the RKI and as part of the diabetes surveillance [20] by USUMA GmbH (Berlin).

The sampling of landline and mobile numbers (dual-frame method) was based on the telephone sampling system provided by the Arbeitskreis Deutscher Markt- und Sozialforschungsinstitute e.V. (ADM), which in principle covers all telephone numbers that can be used in Germany [25]. Further information on the methodology of telephone surveys can be found in a previous publication [26].

A direct screening procedure was used to realize a nationwide sample for adults with diabetes in Germany. For the random selection of the person to be interviewed (i.e. adult person with diabetes), a method developed by Leslie Kish for the random selection of participants in households with several persons was used, the Kish selection grid method [26]. In accordance with the nationwide telephone study Disease Knowledge and Information Needs - Diabetes Mellitus (2017), a sample size of 1,500 participants was targeted [27]. A total of 1,503 adults with physician-diagnosed diabetes participated in the study, who answered the question ‘Have you ever been diagnosed with diabetes by a doctor?’ in the interview. Given the special sampling method used to screen persons with diabetes from the general population, it was not possible to calculate a response rate [28]. For quantification of the study quality, the ratio of the number of completed interviews to the number of started interviews was used instead, which was 81 % in the present study.

**GEDA 2021/2022-Diabetes**

**Data holder:** Robert Koch Institute

**Objectives:** Provision of population-based information on the topics of treatment (immediately after diabetes diagnosis and currently), quality of care (self-management, medical examinations, self-assessed quality of care), subjective general health (currently and compared to the time before the COVID-19 pandemic), mental health (subjective mental health, depressive symptoms, anxiety symptoms) and social risks or resources (social support, loneliness, firm partnership), utilization of outpatient care (GP, specialist or telemedicine services), health behaviour (smoking, physical activity, body weight) and SARS-CoV-2 infection and vaccination in adults with diagnosed diabetes in Germany

**Survey method:** Telephone survey

**Target population:** German-speaking population aged 18 years and over with physician-diagnosed diabetes

**Sampling:** Random sample of landline and mobile phone numbers (dual-frame method) from the ADM (Arbeitskreis Deutscher Markt- und Sozialforschungsinstitute e.V.) sampling system and subsequent screening of persons with diagnosed diabetes in the household

**Sample size:** 1,503 persons with diagnosed diabetes (701 women, 802 men)

**Survey period:** December 2021 – April 2022

**Data protection:** The participants were informed about the objectives and content of the study and data protection and gave their informed consent to participate in the study

More information (in German) at

www.rki.de/geda21-diabetes

As the analysis presented here focuses on the care and health of people with type 2 diabetes, which develops relatively frequently from middle age onwards, participants with type 1 diabetes (n = 40), gestational diabetes (n = 7) and an age under 45 years (n = 8) were excluded, so that the sample on which the data analysis is based comprises 1,448 participants (676 women, 772 men) aged 45 years and over with type 2 diabetes. For the indicator self-assessed quality of care in the last 12 months, participants who did not report having diabetes in the last 12 months were also excluded (n = 58). Sex was collected via self-reported sex on the birth certificate.

### 2.2 Survey content and instruments

#### Behavioural factors, secondary diseases and cardiovascular comorbidities

The body mass index was calculated on the basis of self-reported body weight (in kg) and height (in cm). Smoking of tobacco products, including tobacco heaters, was assessed using the following predefined response options: ‘yes, daily’, ‘yes, occasionally’, ‘no, not more’ and ‘I have never smoked’. The time spent on sport, fitness or physical activity in leisure time in a typical week was assessed using five categories ranging from ‘none’ to ‘5 hours or more per week’.

To collect information on diabetes-related secondary diseases, questions were asked about the presence of the following diseases: ‘diabetes-related kidney disease’, ‘diabetes-related eye disease’, ‘diabetes-related nerve disease’, ‘diabetic foot’ and ‘amputation due to diabetes’ (answer options yes/no in each case).

The ascertainment of cardiovascular diseases in the last 12 months included a ‘heart attack or chronic complaints as a result of a heart attack’, ‘other heart disease’ and a ‘stroke or chronic complaints as a result of a stroke’ (answer options yes/no in each case). To record high blood pressure in the last 12 months, participants were asked about ‘high blood pressure or hypertension, whether treated or untreated’ (answer options yes/no).

Severe hypoglycemia in the last 12 months was determined after confirmation of ‘hypoglycemia’ by asking whether ‘help was needed during such hypoglycemia’, ‘such as from relatives, friends, colleagues, a doctor or other persons’ (answer options yes/no).

#### Healthcare for diabetes

The current treatment of diabetes was documented by asking the following types of treatment: ‘with tablets’, ‘with insulin’, ‘with other blood glucose-lowering medication that is injected (we mean medication to be injected, except insulin)’, ‘with diet or special nutrition’ and ‘with physical activity or sport’ (answer options yes/no in each case). If all types of treatment were denied, the participants were asked ‘Is it correct that your diabetes is not being treated?’ (answer options yes/no). If the answer was yes to treatment with insulin, the participants were also asked whether insulin treatment is carried out using an ‘insulin pump’ or ‘injecting insulin with a pen or syringe’ (answer options yes/no in each case).

The self-management of diabetes was assessed in line with guideline recommendations [29–31] for selected indicators according to their operationalization as part of the diabetes surveillance project [20] on the basis of the following questions: ‘Do you check your feet yourself for pressure sores or open sores?’, ‘Have you ever taken part in a diabetes self-management education programme?’ and ‘Do you - or do family members for you - perform blood glucose self-monitoring?’ (answer options yes/no in each case). If the answer to self-monitoring was yes, the participants were also asked whether they use a blood glucose meter ‘with blood sampling, i.e. by ‘pricking’ the finger or earlobe’ or ‘with a sensor in the subcutaneous fatty tissue, including CGM or flash systems’ (answer options yes/no in each case).

The medical management of diabetes was based on guideline recommendations [7] for selected examinations in the last 12 months according to their mapping in diabetes surveillance [20] using the following questions: ‘When was the last time your HbA1c, i.e. haemoglobin A1c, was determined?’, ‘When was the last time the background of your eyes was examined by an ophthalmologist?’ and ‘When was the last time your feet were examined by a doctor?’ (in each case, how many months or years ago, or ‘never’).

The self-assessment of the quality of care in the last 12 months was carried out using the German version of the diabetes-adapted Patient Assessment of Chronic Illness Care - DAWN short form (PACIC-DSF) based on nine questions (answer options 1 = ‘never’, 2 = ‘rarely’, 3 = ‘sometimes’, 4 = ‘often’, 5 = ‘always’), of which eight questions relate to aspects of patient-oriented care, e.g. the inclusion of personal goals in the treatment plan, and the final question, which measures satisfaction with the organization of treatment [32]. The total score divided by nine represents the PACIC-DSF sum score (scale of 1 – 5). Higher values indicate a better quality of care.

#### Mental health

Self-rated mental health was assessed using the question ‘How would you describe your mental health in general?’ (answer options ‘excellent’, ‘very good’, ‘good’, ‘fair’, ‘poor’) [33, 34]. As this indicator is intended to reflect positive mental health, the proportions of those who rate their mental health as ‘very good’ or ‘excellent’ are reported.

Depressive symptoms were assessed using the internationally established 8-item Patient Health Questionnaire (PHQ-8) based on questions on the frequency of impairment by eight symptoms of depressive disorders in the last two weeks (response options 0 = ‘not at all’, 1 = ‘several days’, 2 = ‘more than half the days’, 3 = ‘nearly every day’) [35]. The presence of depressive symptoms is assumed from a scale sum value of at least 10 (value range 0 – 24).

Anxiety symptoms were assessed by the 2-item Generalized Anxiety Disorder Scale-2 (GAD-2) based on questions about the frequency of impairment by two core symptoms of anxiety disorders in the last two weeks (response options 0 = ‘not at all’, 1 = ‘several days’, 2 = ‘more than half the days’, 3 = ‘nearly every day’) [36]. A considerable burden of anxiety symptoms is assumed from a scale sum value of at least 3 (value range 0 – 6).

#### Social support and loneliness

The perceived availability of social support as a potentially relevant resource for health was measured using the 3-item Oslo Social Support Scale (OSSS-3) based on three questions (scores for the answer options for question 1: 1 to 4, for questions 2 and 3: 1 to 5) [37]. The presence of strong perceived social support is assumed with a scale sum value of at least 12 (value range 3 – 14).

Loneliness as a potential health risk was assessed using the ‘Revised UCLA Loneliness Scale’ (R-UCLA) on the basis of three questions (answer options 1 = ‘rarely or never’, 2 = ‘sometimes’, 3 = ‘often’) [38]. The presence of perceived loneliness is assumed from a scale sum value of at least 6 (value range 3 – 9).

#### Self-assessment of health status

Current subjective health was recorded as part of the Minimum European Health Module with the question ‘How is your health in general?’ (answer options ‘very good’, ‘good’, ‘fair’, ‘bad’, ‘very bad’) [39]. A response of ‘very good’ or ‘good’ is defined as a positive assessment of the general state of health.

The assessment of the change in subjective health compared to the time before the COVID-19 pandemic was assessed with the following question: ‘Compared to the time before the coronavirus pandemic, i.e. before March 2020, how would you describe your current health in general?’ (answer options ‘much better’, ‘slightly better’, ‘about the same’, ‘slightly worse’, ‘much worse’). The proportions of the answers ‘much/slightly better’, ‘about the same’ and ‘much/slightly worse’ were analyzed stratified according to a past SARS-CoV-2 infection. This was assessed with the question ‘Have you ever had an infection with the coronavirus/SARS-CoV-2 detected by a PCR test?’ (answer options yes/no).

### 2.3 Statistical analyses

Deviations in the distribution structure of the sample compared to the population (i.e. German-speaking population aged 18 years and over with physician-diagnosed diabetes) due to a lower willingness to participate can be approximately compensated for with a weighting factor. As the data from the population statistics of the Federal Statistical Office do not allow any conclusions to be drawn about the distribution structure of adults with diagnosed diabetes in the German-speaking population, the weighting was carried out according to the distribution structure of persons with known diabetes from the nationwide telephone survey GEDA 2019/2020-EHIS [40]. For the adjustment, a stepwise weighting was carried out for the characteristics age x sex (in 4 x 2 steps) and age x sex x education (in 2 x 2 x 4 steps) using the SAS software (version 9.4, SAS Institute Inc., Cary, NC, USA).

All results presented were calculated taking into account the corresponding weighting factor. The Stata software (version 17.0, StataCorp LLC, College Station, Texas, USA) was used to calculate the mean values or percentages and 95 % confidence intervals (CI). The results are presented separately for women and men. In addition, differences by age group (45 – 64, 65 – 79, ≥ 80 years), education group (low, medium, high education group; classified according to Comparative Analysis of Social Mobility in Industrial Nations, CASMIN [41]), duration of diabetes (< 5, 5 – 14, ≥ 15 years) and presence of the diabetes-related secondary diseases or cardiovascular comorbidities described under point 2.2 (yes; no, but hypertension; no and no hypertension) are investigated. A statistically significant difference between groups is assumed if the corresponding p-value is less than 0.05 for categorical variables in the Rao-Scott Chi-Square test and for continuous variables in the bivariate linear regression model.

## 3. Results

### 3.1 Characteristics of the study population of persons with type 2 diabetes aged 45 years and over

**Figure 1:**
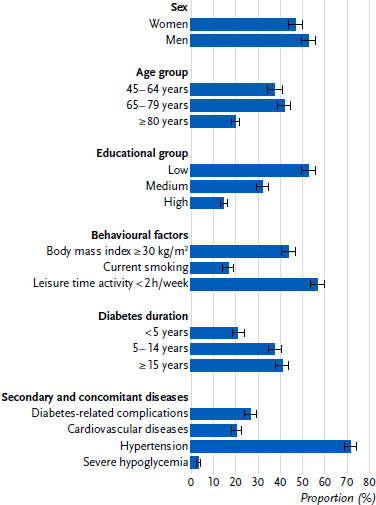
Characteristics (proportions with 95 % confidence interval) of the study population of persons with type 2 diabetes aged 45 years and over (n = 676 women, n = 772 men). Source: GEDA 2021/2022-Diabetes Education group: CASMIN classification [41]; Diabetes-related complications: diabetes-related kidney disease, diabetes-related eye disease, diabetes-related nerve disease, diabetic foot or amputation due to diabetes; Severe hypoglycemia: hypoglycemia in the last 12 months for which help from another person was needed Missing values: n = 2 for education group, n = 20 for diabetes duration, n = 28 for body mass index, n = 2 for smoking, n = 7 for leisure time activity, n = 103 for diabetes-related complications, n = 7 for cardiovascular diseases, n = 3 for hypertension, n = 35 for severe hypoglycemia

The study population comprised women (47.2 %) and men (52.8 %) with type 2 diabetes aged 45 to 99 years, of whom 37.9 % belonged to the age group 45 to 64 years, 42.1 % to the age group 65 to 79 years and 20.0 % to the age group 80 years and over (mean age: 70.3 years (95 % CI 69.7 – 71.0 years)). Of the participants, 52.9 % can be assigned to the low, 32.2 % to the medium and 14.8 % to the high education group. A total of 44.1 % of participants are obese according to their body mass index, 16.8 % smoke daily or occasionally and 57.1 % are physically active for less than 2 hours per week in their leisure time (Figure 1).

41.1 % of participants have diabetes for at least 15 years and 37.7 % for 5 to 14 years, while 21.2 % report diabetes for less than 5 years (mean diabetes duration: 14.0 years (95 % CI 13.3 – 14.7 years)). Diabetes-related secondary diseases are reported by 26.7 % of participants. In addition to diabetes, 72.0 % have high blood pressure and 20.4 % have cardiovascular diseases. Severe hypoglycemia in the last 12 months is reported by 3.4 % of participants (Figure 1).

The sex-stratified analysis shows a higher proportion of women aged 80 years and over, a lower proportion of women in the highly educated group and a lower proportion of women with a leisure time activity of at least 2 hours per week compared to men (Annex Table 1).

### 3.2 Healthcare for type 2 diabetes

#### Treatment

**Figure 2:**
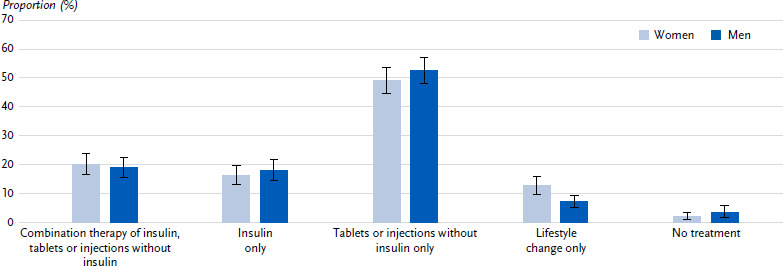
Type of treatment (proportions with 95 % confidence interval) for type 2 diabetes in persons aged 45 years and over by sex (n = 676 women, n = 772 men). Source: GEDA 2021/2022-Diabetes Missing values for treatment type: n = 13

87.5 % of participants (women: 85.2 %, men: 89.3 %, p = 0.076) state that they are receiving pharmacological treatment for their diabetes. Of these, 17.1 % (women: 16.1 %, men: 18.0 %, p = 0.475) receive only insulin, 50.9 % (women: 49.0 %, men: 52.5 %, p = 0.302) only other blood glucose-lowering medication (tablets or injections without insulin) and 19.4 % (women: 20.1 %, men: 18.8 %, p = 0.642) a combination therapy of insulin and other antidiabetics. Treatment solely through changes of dietary behaviour or physical activity is reported by 9.7 % (women: 12.7 %, men: 7.2 %, p = 0.004). A total of 2.8 % (women: 2.1 %, men: 3.5 %, p = 0.250) state that they are currently being treated neither with medication nor by changing their diet or physical activity (Figure 2).

36.5 % (women: 36.2 %, men: 36.8 %, p = 0.858) are treated with insulin (alone or in combination with other antidiabetics), with 35.9 % (women: 35.1 %, men: 36.5 %, p = 0.662) using a syringe or pen and 0.7 % (women: 0.9 %, men: 0.5 %, p = 0.410) using an insulin pump. 8.2 % (women: 9.1 %; men: 7.5 %, p = 0.396) are treated with other blood glucose-lowering medications that are injected (alone or in combination with other antidiabetics).

The further stratified analysis shows no age- and education-related differences. However, as the duration of diabetes increases, the proportion of persons receiving combination therapy with insulin or insulin alone increases, while the proportion of other types of treatment decreases. In the presence of diabetes-related secondary diseases or concomitant cardiovascular diseases, insulin injection only is more frequent and treatment with other antidiabetics only is less frequent than in the presence of hypertension alone (Annex Table 2).

#### Self-management

**Figure 3:**
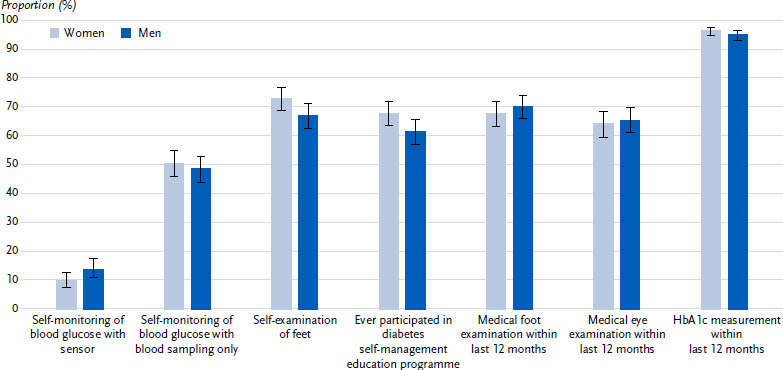
Self-management and medical examinations (proportions with 95 % confidence interval) of persons with type 2 diabetes aged 45 years and over by sex (n = 676 women, n = 772 men). Source: GEDA 2021/2022-Diabetes Missing values: n = 4 for type of blood glucose control, n = 5 for foot self-examination, n = 2 for diabetes education, n = 66 for medical foot examination, n = 27 for medical eye examination, n = 123 for HbA1c measurement

Overall, 62.4 % of participants with type 2 diabetes (women: 61.3 %, men: 63.4 %, p = 0.530) have their blood glucose levels checked by themselves or their family members. Of these, 49.4 % (women: 50.4 %, men: 48.5 %, p = 0.580) monitor their blood glucose levels solely by taking blood samples and 12.0 % (women: 9.8 %, men: 13.9 %, p = 0.062) by continuous glucose monitoring using a sensor in the subcutaneous fatty tissue (Figure 3) (1.0 % did not specify the method). Around a quarter of participants with a sensor (2.9 % in total, women: 2.9 %, men 2.9 %) also check their blood glucose by taking a blood sample.

69.7 % of participants (women: 72.8 %, men: 66.9 %, p = 0.061) state that they check their feet themselves for pressure sores or open sores. 64.3 % (women: 67.7 %, men: 61.3 %, p = 0.045) report having taken part in a diabetes self-management education programme (Figure 3).

The stratified analysis shows more frequent participation in diabetes education among 45- to 79-year-olds compared to older persons. Participants in the middle education group were more likely to report foot self-examination than participants in the low education group. With increasing duration of diabetes, a higher proportion of participants with blood glucose self-monitoring overall, diabetes education and foot self-examination can be observed. In the presence compared to the absence of secondary diseases or cardiovascular comorbidities, a higher proportion of participants with sensor-based blood glucose monitoring and diabetes education can be seen (Annex Table 3).

#### Medical examination

Within the past 12 months, 68.9 % (women: 67.6 %, men: 70.1 %, p = 0.432) of participants had their feet examined by a doctor and 64.8 % (women: 64.1 %, men: 65.4 %, p = 0.676) had their eyes examined. A measurement of the long-term blood glucose value HbA1c in the last 12 months is reported by 95.7 % (women: 96.5 %, men: 95.0 %, p = 0.233) (Figure 3).

A higher proportion of participants with a medical foot examination can be observed with decreasing educational status and increasing duration of diabetes as well as with the presence compared to the absence of secondary or concomitant cardiovascular diseases. The proportion of participants with a medical eye examination is higher in the presence compared to the absence of secondary or cardiovascular diseases and in the age groups 65 years and over compared to the younger age group. In contrast, the proportion of participants with an HbA1c measurement decreases with increasing age (Annex Table 3).

#### Self-assessment of the quality of care

Based on the mean PACIC-DSF sum score (scale from 1 to 5) of 2.37 (women: 2.29, men: 2.45, p = 0.024), participants rate the quality of care for their type 2 diabetes in the last 12 months as moderate on average (Annex Table 4).

The stratified analysis shows a lower mean sum score for the assessment of the quality of care in the age group 80 years and over than in the younger age groups and a higher score the longer the diabetes has been present (Annex Table 4).

### 3.3 Mental health

**Figure 4:**
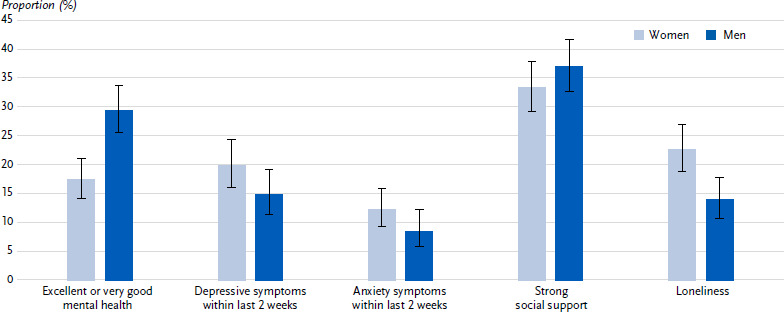
Indicators of mental health, social support and loneliness (proportions with 95 % confidence interval) in persons with type 2 diabetes aged 45 years and over by sex (n = 676 women, n = 772 men). Source: GEDA 2021/2022-Diabetes Missing values: n = 4 for self-assessed mental health, n = 100 for depressive symptoms, n = 25 for anxiety symptoms, n = 64 for social support, n = 24 for loneliness

Of the participants with type 2 diabetes, 23.8 % report very good or excellent mental health in general (women: 17.4 %, men: 29.4 %, p < 0.001). With regard to the last two weeks, 17.2 % (women: 19.9 %, men: 14.8 %, p = 0.087) had depressive symptoms and 10.2 % (women: 12.2 %, men: 8.4 %, p = 0.123) experienced considerable burden from anxiety symptoms (Figure 4).

The proportion of participants with very good or excellent mental health decreases with decreasing educational level and in the presence compared to the absence of diabetes-related secondary diseases or cardiovascular comorbidities. The proportion of participants with with symptoms of depression or anxiety is higher in the low and medium education groups than in the high education group and is highest in the youngest age group (45 – 64 years) (Annex Table 5).

### 3.4 Social support and loneliness

Of the participants, 35.3 % (women: 33.4 %, men: 37.0 %, p = 0.278) perceive strong social support. 18.0 % (women: 22.6 %, men: 13.9 %, p = 0.002) feel lonely (Figure 4).

The further stratified analysis reveals a more frequent feeling of loneliness among 45- to 64-year-olds than among older persons and among participants in the low education group than among participants in the high education group. There are no differences in perceived social support with regard to the characteristics considered (Annex Table 5).

### 3.5 Self-assessment of health status

#### Current self-assessed health

In total, 50.2 % of participants state very good or good health in general (women: 48.1 %, men: 52.2 %, p = 0.228).

The stratified analysis shows lower proportions of very good or good health in the low and medium compared to the high education group as well as in the presence compared to the absence of diabetes-related secondary diseases or cardiovascular comorbidities (Annex Table 6).

#### Change in self-assessed health compared to the time before the COVID-19 pandemic

Compared to the time before the pandemic, 23.9 % of participants (women: 24.5 %, men: 23.4 %, p = 0.700) rate their current general state of health as slightly or much worse, 68.0 % (women: 67.1 %, men: 68.8 %, p = 0.595) as about the same and 8.1 % (women: 8.4 %, men: 7.8 %, p = 0.763) as slightly or much better.

The stratified results indicate that 45- to 64-year-olds compared to 65- to 79-year-olds and participants with diabetes-related secondary diseases or cardiovascular comorbidities compared to those without these diseases are more likely to rate their health as slightly or much worse than before the pandemic (Annex Table 6).

#### Change in self-assessed health compared to the time before the COVID-19 pandemic in people with and without SARS-CoV-2 infection

**Figure 5:**
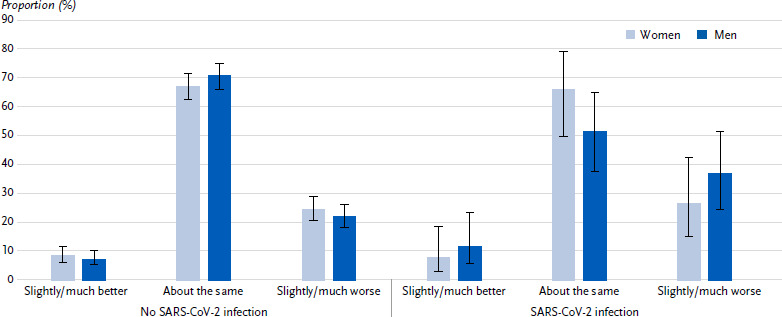
Self-assessed change in health status compared to the time before the COVID-19 pandemic (proportions with 95 % confidence interval) of persons with type 2 diabetes aged 45 years and over by SARS-CoV-2 infection and sex (n = 676 women, n = 772 men). Source: GEDA 2021/2022-Diabetes Missing values: n = 7 for self-assessed health compared to the time before the pandemic, n = 2 for SARS-CoV-2 infection

At the time of the interview, almost a tenth of participants reported that they had already been diagnosed with a SARS-CoV-2 infection (9.1 %, women: 8.2 %, men: 10.0 %, p = 0.351). Among participants with a SARS-CoV-2 infection, a higher proportion with health rated as worse than before the pandemic can be observed compared to those without an infection (32.5 % vs. 23.1 %), with the difference being particularly pronounced among men (women: 26.4 % vs. 24.4 %, men: 36.9 % vs. 21.9 %). Accordingly, there is a lower proportion of participants with an infection compared to those without an infection with health rated as about the same than before the pandemic (57.4 % vs. 69.0 %), which is again particularly attributable to men (women: 65.9 % vs. 67.1 %, men: 51.3 % vs. 70.7 %). The proportion of participants with health rated as better than before the pandemic is similar for those with and without an infection (10.1 % vs. 7.9 %, women: 7.7 % vs. 8.4 %, men: 11.8 % vs. 7.3 %) (Figure 5).

Further analysis shows that the differences observed between participants with and without SARS-CoV-2 infection are particularly pronounced in the middle age group of 65- to 79-year-olds (Annex Table 7).

## 4. Discussion

The study GEDA 2021/2022-Diabetes was conducted as part of diabetes surveillance [20] and is a nationwide, population-based cross-sectional survey of adults with diagnosed diabetes in a period during the end of the COVID-19 pandemic [21]. This present analysis considers key indicators from different fields of action in people aged 45 years and over with type 2 diabetes, both overall and stratified by socio-demographic and disease-related characteristics, in order to obtain information on potential vulnerable groups. The use of established questions and instruments enables the comparison over time with previous survey data of people with diabetes as well as the comparison with data collected at the same time from the general population in Germany.

### 4.1 Healthcare for type 2 diabetes

#### Treatment

In conjunction with data from examination surveys, the proportion of people aged 45 years and over with type 2 diabetes, who are not treated with medication or lifestyle changes, has decreased (German National Health Interview and Examination Survey 1998 (GNHIES98): 13.6 %; German Health Interview and Examination Survey for Adults 2008 – 2011 (DEGS1): 17.3 %; current study: 2.8 %) [42]. It should be noted that, unlike the earlier analyses, the current analysis also includes people aged 80 years and over and excludes women with a previous gestational diabetes, which may partly explain the current higher proportion of 87.5 % with pharmacological treatment [43]. An increase in the proportion of adults with type 2 diabetes receiving blood glucose-lowering medication was also shown in an analysis of registry data from Germany and Austria between 2002 and 2014 (64.0 % vs. 78.2 %) [44]. A similar proportion of pharmacological therapy in adults with type 2 diabetes for the period 2012 to 2014 was estimated based on routine data (72.4 %) [45]. Based on registry data, there was a further increase in pharmacological treatment for type 2 diabetes until 2021 [46]. Compared to the examination surveys, an increase in the combination therapy of insulin with other antidiabetics can be observed (GNHIES98: 8.0 %; DEGS1: 13.6 %; current study: 19.4 %) [42]. According to the National Treatment Guideline, there are indications of advantages of combination therapy of insulin with other antidiabetics, so that, for example, if the individual therapy goals are not achieved with oral antidiabetics alone, combination therapy with insulin is initially recommended instead of insulin therapy alone [7]. As expected, the proportion of people on insulin therapy increases with an increasing duration of diabetes and the occurrence of complications [47–49]. The low proportion of insulin pump therapy in people with type 2 diabetes (0.7 %) is in line with the results of an study of diabetologists in 2023 regarding new technologies in their facilities, according to which a pump is only used in around 0.4 % of people with type 2 diabetes [50]. According to the guideline, pump therapy is rarely indicated for type 2 diabetes [7] and the effectiveness of this form of treatment compared to others still needs to be investigated for type 2 diabetes [51].

As previously in DEGS1, the present study shows no differences in treatment according to age or education [52]. Sex differences in the current study are limited to a more frequent treatment solely by lifestyle changes in women than in men, which was already found as a tendency based on DEGS1 data [53]. This could be related to the obeservation that women are more likely to have weight gain or obesity at the time of diagnosis compared to men; however, women are also more likely to be undertreated with medication [53, 54].

#### Self-management and medical examination

Of the indicators in this area, which were defined in accordance with guideline recommendations and analogous to their use in the diabetes surveillance [20], participants aged 45 years and over with type 2 diabetes most frequently reported a HbA1c measurement in the last 12 months (95.7 %). This proportion was similarly high (93.2 %) among 45- to 79-year-olds with type 2 diabetes based on DEGS1 data [53, 55]. This proportion seems plausible, as according to the disease management program (DMP) guideline HbA1c measurements to monitor long-term blood glucose levels should even be carried out quarterly, but at least every six months [56]. A comparison of the current results on medical foot and eye examinations (68.9 % and 64.8 % respectively) with results from DEGS1 for 45- to 79-year-olds with type 2 diabetes (61.4 % and 78.4 % respectively) and GEDA 2014/2015-EHIS for people aged 18 years and over with diabetes in the last 12 months (around 62 % and 76 % respectively) indicates an increase in annual foot examinations and a decrease in annual eye examinations [42, 57]. Regular examinations serve to detect and treat diabetes-related foot lesions and eye diseases at an early stage [7]. The decline in eye examinations could at least partly reflect an adjustment to the guidelines, according to which an examination has only been recommended every one to two years since 2015, depending on the risk profile and retinal changes [7, 58]. However, based on data from adult participants in the DMP type 2 diabetes in North Rhine-Westphalia, a decline from 73.3 % in 2017 to 64.1 % in 2021 can also be observed in relation to retinal examinations in the last 24 months [59]. Based on a study with DMP data from an urban region in Hesse in 2019, there are also indications that less than half of the participants were fully screened for retinal changes in accordance with the German guidelines, including a written report to the referring practice [60].

The comparison of the present results for foot self-examination (69.7 %) and participation in a diabetes self-management education programme (64.3 %) with results from GEDA 2014/2015-EHIS (around 71 % and 63 % respectively) shows similar proportions at both points in time [57]. Similarly, the proportion of those who monitor their blood glucose level themselves or with the help of their family members (62.4 %) is similar when compared with earlier data from DEGS1 (62.8 %) and GEDA 2014/2015-EHIS (around 66 %) [42, 57]. Blood glucose self-monitoring, which supports the achievement of individual blood glucose therapy goals and the avoidance of acute hypo- and hyperglycaemia, can be facilitated by continuous glucose monitoring with measurement of the ‘tissue glucose’ in the subcutaneous fatty tissue by a sensor and is mainly used by people on insulin therapy. As expected, the proportion of sensor-based blood glucose monitoring in participants with type 2 diabetes in this study (12.0 %) is considerably lower than in the study of diabetologists in 2023 for people with insulin-dependent type 1 diabetes (77.3 %) [50] or on the basis of registry data for 2019 for children and adolescents with insulin-dependent type 1 diabetes (69.3 %) [61]. However, the corresponding proportion for people with type 2 diabetes according to registry data for the age group 60 years and over in 2021 (18 % [62]) and according to the study of diabetologists in 2023 (22.4 % [50]) are also slightly higher than in the present study, but this might be due to the increasing proportion over time [50, 62] or to the different study design.

With regard to socio-demographic characteristics, only slight differences can be observed in the proportions of the indicators considered in this study. These mainly include less frequent participation in diabetes self-management education programmes for people aged 80 years and over and less frequent ophthalmologic examinations for people under 65 years compared to the other age groups as well as more frequent medical foot examinations with decreasing educational level. In contrast, there are clear differences for most self-examinations and medical check-ups with regard to the duration of diabetes and the presence of secondary and concomitant diseases. In particular, the proportion of participants with diabetes for less than 5 years who participated in diabetes eductation programmes is almost half that of those with diabetes for 15 years or more (40.1 % vs. 76.5 %). However, as diabetes education has been described as the most important predictor of good diabetes self-management [63], measures to encourage participation in diabetes education programmes at an early stage of the disease could potentially help to prevent or delay long-term consequences and adverse psychosocial aspects of the disease burden.

#### Self-assessment of the quality of care

The result of the present study on the PACIC-DSF score (2.37 on a scale of 1 – 5) shows that compared to the result of the score used in identical form in the study ‘Disease knowledge and information needs - diabetes mellitus (2017)’ (2.47) [32] people with type 2 diabetes continue to rate the overall quality of care for their disease in the last 12 months as only moderate. While no change can be observed in women (2.29 vs. 2.33), there is an indication of a slight deterioration in the self-assessment of the quality of care for men (2.45 vs. 2.58) [32]. Nevertheless, men continue to assess their quality of care as slightly better than women.

In addition, the quality of care is rated as poorer with increasing age and shorter duration of diabetes. A greater need for care and higher psychosocial load due to increasing health problems with increasing age could possibly have contributed to the age-related difference in the self-assessed quality of care. A longer duration of diabetes could facilitate dealing with type 2 diabetes and therefore the quality of care could be assessed better than with a shorter duration of diabetes.

The quality of care assessed as moderate by the participants probably relates less to the assessment of the organization of care, but rather to the integration of the participants’ needs (e.g. with regard to support in self-management and in achieving treatment goals as well as with regard to the tolerability of the therapy) [32]. Further research in this area is necessary in order to better understand the assessment of the quality of care from the perspective of those affected and to be able to derive suitable measures to improve care.

### 4.2 Mental health

The presented study results can be partially compared with results for the same age group from the general population based on the GEDA study [40], which were published on a dashboard as part of a continuous evaluation of the Mental Health Surveillance at the RKI [64]. Looking at results from the time period that is also covered in the present study, the proportion of people with very good or excellent self-rated mental health is lower among adults with type 2 diabetes than in the general population (particularly for 45- to 64-year-olds: 24.0 % vs. 38 % – 39 %). However, there is no difference in the proportions with anxiety symptoms. For depressive symptoms, no results for the general population for the observation period reported here are available that could be used for comparison. However, in the earlier studies GEDA 2014/2015-EHIS and GEDA 2019/2020-EHIS, a higher prevalence of depressive symptoms measured using the PHQ-8 was observed in people aged 18 years and over with diabetes than in those without diabetes [65].

A comparison of the present results with pre-pandemic data from people with type 2 diabetes is only possible for depressive symptoms according to screening with the PHQ-8 [65], but not for the other two indicators of mental health. A direct comparison of the estimates from GEDA 2014/2015-EHIS and GEDA 2019/2020-EHIS with the present study indicates an increase in depressive symptoms (45- to 64-year-olds: 19.5 % vs. 17.7 % vs. 26.5 %; 65- to 79-year-olds: 8.7 % vs. 7.9 % vs. 12.3 %), albeit with overlaps in the confidence intervals. In an analysis of GEDA data between 2019/2020 and 2021/2022, an increase in depressive symptoms was observed for people aged 45 years and over in the general population according to screening with the shorter PHQ-2 [66]. An increase is therefore also plausible for people with type 2 diabetes.

The lower proportion of self-rated mental health as very good or excellent and the more frequent presence of depressive and anxiety symptoms in the low compared to the high education group are consistent with results from the general population aged 18 years and over. This also applies to the observation that women are less likely than men to self-assess their mental health as excellent or very good [66]. In addition, depressive and anxiety symptoms are also more common in the general population aged 45 to 64 years than in older people [67].

### 4.3 Social support and loneliness

A comparison of the present results with the results of the Mental Health Surveillance for the general population for the same obvservation period [67] shows that neither perceived social support nor perceived loneliness among participants with type 2 diabetes are to be considered noticeable.

Similar to the general population, people with type 2 diabetes were more likely to experience loneliness in younger than in older age, in women than in men and in the low compared to the high education group [67]. In contrast, the sex- and education-related differences in perceived social support that were found in the general population [67] were not found in participants with type 2 diabetes at the time of observation of the current study.

### 4.4 Self-assessment of health status

#### Current self-assessed health

In GEDA 2019/2020-EHIS, a higher proportion of people aged 45 – 64 years in the general population rated their health as very good or good (women: 66.0 %, men: 65.2 %) [68] than people with type 2 diabetes in the present study (overall: 46.1 %). This difference is less pronounced in the older age groups, which could be due to the increasing burden of disease in the general population with increasing age and the associated poorer assessment of health.

Lower proportions with health rated as very good or good in the low and medium compared to the high education group are shown both for people with type 2 diabetes in the current study and in the general population based on GEDA 2019/2020-EHIS [68]. In addition, the present study shows a considerably lower proportion with health rated as very good or good in the presence compared to the absence (35.1 % vs. 67.6 %) of secondary diseases and cardiovascular comorbidities.

#### Change in self-rated health compared to the time before the COVID-19 pandemic

The European SHARE study showed that between mid-2020 and mid-2021, the proportion of people aged 50 years and over in the general population who assessed their health as worse than around three months previously increased (28 countries in total: 8.7 % vs. 14.5 %, Germany: 8.9 % vs. 13.4 %) [69]. In the CORONA-MONITORING local – Follow-up study, adults with a SARS-CoV-2 infection in 2020 more frequently reported only moderate to very poor subjective health after more than one year than people without an infection (19.3 % vs. 13.0 %) [24]. Own unpublished analyses of the GEDA 2022 survey [70] (wave 2: 09.02. – 09.04.2022) with identical recording of changes in health and previous SARS-CoV-2 infections result in a self-assessed deterioration in health in 20.5 % of non-infected and 23.1 % of infected persons aged 45 years and over in the general population. In the present study, for participants with type 2 diabetes, the proportion with a reported health deterioration is similar among non-infected persons (23.1 %), but higher among infected persons (32.5 %), especially for the age group 65 to 79 years and for men. This observation indicates that people with diabetes are more likely to perceive a deterioration in their health compared to the pre-pandemic period if they have already had a SARS-CoV-2 infection, with older people and men being particularly affected. This could be related to indirect and direct effects of the COVID-19 pandemic, e.g. delayed utilization of healthcare and nursing services [71, 72] and the mutually unfavorable influencing course of diabetes and COVID-19 [73].

### 4.5 Limitations

The limitations of this study are mainly due to the data collection using a telephone survey. Telephone interviews can lead to socially desirable response behaviour and thus to an over- or underestimation of the actual prevalence of potentially sensitive topics [74]. In addition, telephone surveys often show that people in the lower education group or in older age (especially seriously ill people or people living in care facilities) participate less frequently, which means that the proportion of these groups in the sample differs from that in the population. Therefore, despite the established sampling and screening method [25, 26] and the weighting procedure described, a bias due to the selection of participants (selection bias) cannot be ruled out. In addition, only German-speaking interviews were conducted. As a result, people with little or no knowledge of German were not included in the study population. Furthermore, the data was self-reported by the participants, thus incorrect assessments, e.g. regarding diabetes-related complications, wich are to be distinguished from non-diabetes-related complications, cannot be ruled out.

In the present study, measurement and laboratory data-based indicators such as the achievement of specific treatment targets for blood pressure, HbA1c and cholesterol were not obtained. For surveillance of corresponding indicators, which are also defined as relevant core indicators in the context of the diabetes surveillance at the RKI [20], regular nationwide, population-based examination studies need to be established.

The interpretation of the calculated indicators for participants with type 2 diabetes is limited to the comparison, where possible, with earlier results on the healthcare situation of people with type 2 diabetes or with existing results on mental and general health as well as social integration from the general population in Germany. A more extensive comparison with other European countries is only possible with difficulty due to differences in the operationalization of the indicators and differences in the underlying study populations and healthcare systems.

### 4.6 Conclusion

Almost nine out of ten participants with type 2 diabetes aged 45 years and over are being treated with antidiabetics. Around one in three receive insulin therapy or a combination therapy with insulin and other antidiabetics. Although the longterm blood glucose value HbA1c is measured at least once a year in almost all those affected, annual medical foot and eye examinations, participation in a diabetes self-management education programme and blood glucose and foot self-monitoring are still only reported by around two thirds. This observation is consistent with the result that those affected rate the quality of care for their diabetes as moderate, which has remained relatively unchanged over the last few years, and points to a clear potential for improvement in care. It is particularly noteworthy that people with a relatively short duration of diabetes have the least favorable values in terms of self-management and perceived quality of care.

This study shows that in middle adulthood, only almost a quarter of participants with type 2 diabetes rate their mental health as excellent or very good, compared to more than a third in the general population. In this age range, there are also indications of an increase in depressive symptoms compared to earlier data, which is observed in the present study for around a quarter of 45- to 64-year-olds with type 2 diabetes. It needs to be emphasized that there are education-related differences for self-rated mental health and the symptoms considered, to the disadvantage of the low education group. The results are consistent with calls for attention to the possible presence of clinical or subclinical depression when treating people with diabetes [12, 75].

The general health of people with type 2 diabetes who have had a SARS-CoV-2 infection, which is more frequently rated to be worse than before the pandemic, requires special attention in the care process.

## Key statement

- Of participants aged 45 years and over with type 2 diabetes, 26.7 % have diabetes-related secondary diseases, 72.0 % have hypertension and 20.4 % have cardiovascular diseases.
- A total of 87.5 % are treated with medication – 36.5 % with insulin alone or in combination with other antidiabetics.
- Over 90 % have HbA1c measurements and over 60 % report medical examinations of feet and eyes, diabetes self-management education, and self-monitoring of feet and blood glucose.
- 23.8 % have excellent/very good subjective mental health, and 35.3 % have strong social support.
- 23.9 % report a deterioration of general health compared to the time before the pandemic, in particular with previous SARS-CoV-2 infection.

## Figures and Tables

**Annex Table 1:** Characteristics of the study population of participants with type 2 diabetes aged 45 years and over by sex (n = 676 women, n = 772 men). Source: GEDA 2021/2022-Diabetes

	Women		Men	
	%	(95 % CI)	%	(95 % CI)
**Age group**				
45 – 64 years	31.6	(27.0 – 36.7)	43.5	(38.6 – 48.4)
65 – 79 years	44.7	(40.1 – 49.3)	39.7	(35.6 – 44.1)
≥ 80 years	23.7	(20.3 – 27.5)	16.8	(14.2 – 19.7)
**Education group**				
Low	60.5	(56.1 – 64.8)	46.1	(41.4 – 51.0)
Medium	32.8	(28.9 – 37.0)	31.7	(27.7 – 36.0)
High	6.6	(5.4 – 8.2)	22.2	(19.2 – 25.4)
**Behavioural factors**				
Body mass index ≥ 30 kg/m^2^	43.9	(39.2 – 48.8)	44.3	(39.7 – 49.1)
Current smoking	14.8	(11 .4 – 19.0)	18.5	(14.9 – 22.8)
Leisure time activity < 2 h/week	61.4	(56.7 – 65.8)	53.3	(48.6 – 57.9)
**Diabetes duration**				
< 5 years	22.5	(18.7 – 26.9)	20.0	(16.4 – 24.2)
5 – 14 years	36.0	(31.6 – 40.7)	39.3	(34.7 – 44.0)
≥ 15 years	41.5	(36.9 – 46.2)	40.7	(36.3 – 45.3)
**Secondary and concomitant diseases**				
Diabetes-related complications	25.4	(21.4 – 29.9)	27.8	(23.8 – 32.2)
Cardiovascular diseases	20.6	(17.1 – 24.5)	20.2	(17.2 – 23.7)
Hypertension	72.5	(68.1 – 76.6)	71.6	(67.3 – 75.5)
Severe hypoglycemia	3.4	(2.0 – 5.6)	3.4	(2.1 – 5.5)
**Total**	**47.2**	**(43.9 – 50.5)**	**52.8**	**(49.5 – 56.5)**

Education group: CASMIN classification [41]; Diabetes-related complications: diabetes-related kidney disease, diabetes-related eye disease, diabetes-related nerve disease, diabetic foot or amputation due to diabetes; Severe hypoglycemia: hypoglycemia in the last 12 months for which help from another person was needed

Missing values: n = 2 for education group, n = 20 for diabetes duration, n = 28 for body mass index, n = 2 for smoking, n = 7 for leisure time activity, n = 103 for diabetes-related complications, n = 7 for cardiovascular diseases, n = 3 for hypertension, n = 35 for severe hypoglycemia

**Annex Table 2:** Type of treatment for type 2 diabetes in persons aged 45 years and over by age group, education group, duration of diabetes and presence of secondary and concomitant diseases (n = 676 women, n = 772 men). Source: GEDA 2021/2022-Diabetes

	Combination therapy of insulin, tablets or injections without insulin		Insulin only		Tablets or injections without insulin		Lifestyle change only		No treatment	
	%	(95 % CI)	%	(95 % CI)	%	(95 % CI)	%	(95 % CI)	%	(95 % CI)
**Age group**										
45 – 64 years	19.2	(14.6 – 24.9)	16.4	(12.0 – 22.0)	51.2	(44.6 – 57.7)	9.0	(6.0 – 13.4)	4.1^*^	(2.0 – 8.4)
65 – 79 years	22.1	(18.6 – 25.9)	16.2	(13.1 – 19.8)	51.6	(47.2 – 56.0)	8.6	(6.4 – 11.4)	1.5	(0.7 – 3.0)
≥ 80 years	14.3	(10.6 – 18.9)	20.3	(15.8 – 25.6)	48.7	(42.7 – 54.6)	13.3	(9.8 – 17.9)	3.3	(1.7 – 6.2)
**Education group**										
Low	19.1	(15.3 – 23.5)	18.3	(14.7 – 22.7)	48.6	(43.4 – 53.9)	10.2	(7.5 – 13.7)	3.3	(1.7 – 6.2)
Medium	22.1	(18.1 – 26.7)	16.6	(13.0 – 21.0)	51.6	(46.4 – 56.7)	7.3	(5.2 – 10.2)	3.6	(1.9 – 6.6)
High	15.6	(11.7 – 20.5)	13.5	(10.0 – 18.1)	56.2	(50.3 – 62.0)	13.4	(9.8 – 18.1)	2.4^*^	(1.2 – 4.7)
**Diabetes duration**										
< 5 years	10.1	(6.2 – 15.9)	8.7	(5.1 – 14.4)	62.6	(54.9 – 69.6)	15.7	(11.1 – 21.8)	2.9^*^	(1.2 – 6.8)
5 – 14 years	16.9	(13.1 – 21.5)	12.3	(8.8 – 16.9)	57.8	(52.1 – 63.3)	9.6	(7.0 – 13.1)	3.3	(1.6 – 7.0)
≥ 15 years	26.7	(22.5 – 31.4)	26.3	(22.1 – 30.9)	38.6	(34.0 – 43.5)	6.0	(4.0 – 8.9)	2.3	(1.1 – 4.9)
**Diabetes-related complications and cardiovascular diseases**										
Yes	21.5	(17.6 – 26.0)	24.8	(20.4 – 29.7)	42.5	(37.6 – 47.6)	8.4	(6.1 – 11.4)	2.8	(1.5 – 5.4)
No, but hypertension	20.8	(16.5 – 25.8)	9.1	(6.5 – 12.5)	58.6	(52.9 – 64.0)	10.0	(7.1 – 13.9)	1.5^*^	(0.5 – 4.5)
No, and no hypertension	15.5	(10.7 – 21.8)	16.8	(11 .3 – 24.2)	50.6	(42.8 – 58.5)	12.5	(8.0 – 19.0)	4.6^*^	(2.0 – 10.1)
**Total**	**19.4**	**(16.9 – 22.2)**	**17.1**	**(14.7 – 19.8)**	**50.9**	**(47.5 – 54.2)**	**9.7**	**(7.9 – 11.8)**	**2.8**	**(1.8 – 4.4)**

Education group: CASMIN classification [41]; diabetes-related complications: diabetes-related kidney disease, diabetes-related eye disease, diabetes-related nerve disease, diabetic foot or amputation due to diabetes

^*^Number of cases is n < 10; missing values for treatment type: n = 13

**Annex Table 3:** Self-management and medical examinations of persons with type 2 diabetes aged 45 years and over by age group, education group, duration of diabetes and presence of secondary and concomitant diseases (n = 676 women, n = 772 men). Source: GEDA 2021/2022-Diabetes

	Self-monitoring of blood glucose with sensor		Self-monitoring of blood glucose with blood sampling only		Self-examination of feet		Ever participated in diabetes self-management education programme		Medical foot examination within last 12 months		Medical eye examination within last 12 months		HbA1c measurement within last 12 months	
	%	(95 % CI)	%	(95 % CI)	%	(95 % CI)	%	(95 % CI)	%	(95 % CI)	%	(95 % CI)	%	(95 % CI)
**Age group**														
45 – 64 years	14.7	(10.0 – 20.0)	46.6	(40.1 – 53.2)	70.3	(63.9 – 76.1)	63.9	(57.3 – 70.1)	66.2	(59.7 – 72.1)	58.2	(51.5 – 64.5)	97.6	(94.7 – 99.0)
65 – 79 years	11.0	(8.5 – 14.0)	51.9	(47.5 – 56.2)	71.8	(67.6 – 75.6)	69.5	(65.5 – 73.3)	72.8	(68.7 – 76.5)	69.1	(64.9 – 73.0)	95.3	(93.3 – 96.8)
≥ 80 years	8.7	(5.9 – 12.7)	49.4	(43.5 – 55.3)	64.0	(58.1 – 69.4)	54.0	(48.1 – 59.9)	65.9	(59.9 – 71.5)	68.4	(62.6 – 73.6)	92.2	(87.8 – 95.2)
**Education group**														
Low	10.0	(7.2 – 13.7)	48.5	(43.4 – 53.7)	67.0	(61.9 – 71.7)	66.0	(60.9 – 70.7)	72.6	(67.6 – 77.1)	67.1	(61.9 – 71.9)	95.9	(93.4 – 97.5)
Medium	15.0	(11.5 – 19.4)	50.3	(45.1 – 55.5)	75.3	(70.6 – 79.4)	62.6	(57.5 – 67.5)	65.8	(60.6 – 70.7)	63.4	(58.3 – 68.3)	96.2	(94.0 – 97.6)
High	10.7	(7.5 – 14.9)	51.8	(45.9 – 57.6)	69.2	(63.5 – 74.3)	62.2	(56.4 – 67.8)	61.4	(55.3 – 67.2)	60.1	(54.1 – 65.8)	93.6	(90.5 – 95.7)
**Diabetes duration**														
< 5 years	8.2	(4.7 – 13.9)	38.1	(31.0 – 45.7)	62.0	(54.3 – 69.2)	40.1	(32.9 – 47.7)	54.4	(46.6 – 62.0)	58.6	(50.8 – 66.0)	94.9	(89.8 – 97.6)
5 – 14 years	10.1	(7.2 – 13.9)	50.2	(44.6 – 55.8)	70.2	(64.9 – 75.1)	64.6	(59.1 – 69.7)	68.6	(63.1 – 73.7)	66.2	(60.7 – 71.3)	95.5	(93.2 – 97.0)
≥ 15 years	16.0	(12.5 – 20.2)	54.6	(49.6 – 59.5)	73.4	(68.7 – 77.6)	76.5	(72.3 – 80.3)	76.6	(72.3 – 80.4)	67.0	(62.0 – 71.5)	96.1	(93.9 – 97.5)
**Diabetes-related complications and cardiovascular diseases**														
Yes	17.3	(13.5 – 22.0)	52.9	(47.8 – 58.0)	70.3	(65.4 – 74.8)	70.2	(65.4 – 74.5)	73.7	(69.0 – 77.9)	71.3	(66.6 – 75.6)	96.3	(94.3 – 97.6)
No, but hypertension	7.7	(5.2 – 11.1)	49.4	(43.8 – 55.0)	69.9	(64.5 – 74.8)	60.1	(54.5 – 65.5)	66.7	(61.1 – 71.8)	60.8	(55.1 – 66.3)	95.9	(93.0 – 97.6)
No, and no hypertension	9.1	(5.8 – 13.9)	47.2	(39.4 – 55.1)	74.2	(67.0 – 80.3)	63.5	(55.7 – 70.8)	68.5	(60.8 – 75.4)	59.6	(51.5 – 67.3)	93.6	(89.0 – 96.3)
**Total**	**12.0**	**(9.9 – 14.3)**	**49.4**	**(46.0 – 52.7)**	**69.7**	**(66.5 – 72.7)**	**64.3**	**(61.1 – 67.4)**	**68.9**	**(65.7 – 71.9)**	**64.8**	**(61.5 – 67.9)**	**95.7**	**(94.2 – 96.8)**

Education group: CASMIN classification [41]; diabetes-related complications: diabetes-related kidney disease, diabetes-related eye disease, diabetes-related nerve disease, diabetic foot or amputation due to diabetes

Missing values: n = 4 for blood glucose control, n = 5 for foot self-examination, n = 2 for diabetes education, n = 66 for medical foot examination, n = 27 for medical eye examination, n = 123 for HbA1c measurement

**Annex Table 4:** Self-assessment of the quality of care (n = 650 women, n = 740 men) of persons with type 2 diabetes in the last 12 months aged 45 years and over by age group, education group, duration of diabetes and presence of secondary and concomitant diseases. Source: GEDA 2021/2022-Diabetes

	PACIC-DSF sum score for self-assessed quality of care within last 12 months	
	Mean value	(95 % CI)
**Sex**		
Women	2.29	(2.19 – 2.39)
Men	2.45	(2.35 – 2.54)
**Age group**		
45 – 64 years	2.46	(2.32 – 2.60)
65 – 79 years	2.38	(2.29 – 2.47)
≥ 80 years	2.15	(2.04 – 2.26)
**Education group**		
Low	2.36	(2.25 – 2.47)
Medium	2.38	(2.27 – 2.48)
High	2.37	(2.25 – 2.50)
**Diabetes duration**		
< 5 years	2.16	(1.99 – 2.32)
5 – 14 years	2.38	(2.26 – 2.50)
≥ 15 years	2.48	(2.39 – 2.58)
**Diabetes-related complications and cardiovascular diseases**		
Yes	2.39	(2.29 – 2.50)
No, but hypertension	2.37	(2.26 – 2.48)
No, and no hypertension	2.40	(2.22 – 2.59)
**Total**	**2.37**	**(2.30 – 2.44)**

PACIC-DSF sum score: sum of the answers from nine individual questions (answer options 1 = ‘never’, 2 = ‘rarely’, 3 = ‘sometimes’, 4 = ‘often’, 5 = ‘always’) divided by nine (scale 1 – 5)

Education group: CASMIN classification [41]; diabetes-related complications: diabetes-related kidney disease, diabetes-related eye disease, diabetes-related nerve disease, diabetic foot or amputation due to diabetes

Missing values for self-assessed quality of care: n = 165

**Annex Table 5:** Indicators of mental health, social support and loneliness in persons with type 2 diabetes aged 45 years and over by age group, education group, duration of diabetes and presence of secondary and concomitant diseases (n = 676 women, n = 772 men). Source: GEDA 2021/2022-Diabetes

	Excellent or very good mental health		Depressive symptoms within last 2 weeks		Anxiety symptoms within last 2 weeks		Strong social support		Loneliness	
	%	(95 % CI)	%	(95 % CI)	%	(95 % CI)	%	(95 % CI)	%	(95 % CI)
**Age group**										
45 – 64 years	24.0	(18.9 – 30.0)	26.5	(20.7 – 33.3)	17.0	(12.4 – 23.0)	35.4	(29.3 – 42.0)	23.8	(18.5 – 30.1)
65 – 79 years	24.6	(21.1 – 28.4)	12.3	(9.5 – 15.8)	5.3	(3.5 – 7.9)	36.0	(31.9 – 40.4)	14.4	(11.5 – 17.9)
≥ 80 years	21.5	(17.1 – 26.7)	9.9	(6.8 – 14.1)	7.9	(5.2 – 12.0)	33.5	(27.9 – 39.5)	14.6	(10.9 – 19.4)
**Education group**										
Low	20.6	(16.7 – 25.1)	20.0	(15.7 – 25.1)	12.5	(9.1 – 17.0)	33.0	(28.2 – 38.1)	20.1	(16.1 – 24.9)
Medium	25.3	(21.1 – 30.0)	18.2	(14.2 – 23.0)	9.6	(6.9 – 13.3)	38.1	(33.1 – 43.4)	17.6	(13.9 – 22.1)
High	32.5	(27.5 – 37.9)	5.5	(3.7 – 8.2)	3.7	(2.2 – 6.4)	37.1	(31.7 – 42.9)	10.5	(7.7 – 14.2)
**Diabetes duration**										
< 5 years	26.0	(19.7 – 33.3)	19.7	(13.8 – 27.4)	10.5	(6.3 – 17.1)	39.2	(32.1 – 46.8)	17.0	(11.7 – 24.0)
5 – 14 years	25.0	(20.6 – 29.9)	16.3	(12.0 – 21.9)	11.3	(7.8 – 16.2)	34.0	(28.9 – 39.6)	18.1	(13.9 – 23.3)
≥ 15 years	22.2	(18.5 – 26.4)	16.9	(13.1 – 21.6)	9.2	(6.4 – 13.1)	34.6	(30.0 – 39.6)	17.5	(13.8 – 21.9)
**Diabetes-related complications and cardiovascular diseases**										
Yes	19.1	(15.5 – 23.3)	17.7	(13.9 – 22.3)	10.6	(7.6 – 14.6)	35.1	(30.2 – 40.2)	20.0	(16.0 – 24.7)
No, but hypertension	26.0	(21.5 – 31.1)	15.6	(11.3 – 21.0)	8.2	(5.2 – 12.7)	37.2	(31.9 – 42.8)	16.5	(12.5 – 21.4)
No, and no hypertension	32.6	(25.8 – 40.3)	15.7	(9.9 – 23.9)	9.7	(5.5 – 16.7)	35.2	(28.1 – 43.0)	12.5	(7.9 – 19.3)
**Total**	**23.8**	**(21.1 – 26.6)**	**17.2**	**(14.5 – 20.3)**	**10.2**	**(8.1 – 12 .8)**	**35.3**	**(32.1 – 38.6)**	**18.0**	**(15.4 – 20.9)**

Education group: CASMIN classification [41]; diabetes-related complications: diabetes-related kidney disease, diabetes-related eye disease, diabetes-related nerve disease, diabetic foot or amputation due to diabetes

Missing values: n = 4 for self-assessed mental health, n = 100 for depressive symptoms, n = 25 for anxiety symptoms, n = 64 for social support, n = 24 for loneliness

**Annex Table 6:** Self-assessed health status and change in health status compared to the time before the COVID-19 pandemic of persons with type 2 diabetes aged 45 years and over by age group, education group, duration of diabetes and presence of secondary and concomitant diseases (n = 676 women, n = 772 men). Source: GEDA 2021/2022-Diabetes

	Self-assessed general health		Self-assessed general health compared to the time before the COVID-19 pandemic					
	Very good/good		Much/slightly better		About the same		Much/slightly worse	
	%	(95 % CI)	%	(95 % CI)	%	(95 % CI)	%	(95 % CI)
**Sex**								
Women	48.1	(43.4 – 52.8)	8.4	(6.0 – 11 .5)	67.1	(62.5 – 71.4)	24.5	(20.7 – 28.8)
Men	52.2	(47.5 – 56.8)	7.8	(5.7 – 10.6)	68.8	(64.2 – 73.1)	23.4	(19.5 – 27.8)
**Age group**								
45 – 64 years	46.1	(39.6 – 52.7)	10.4	(7.1 – 15.1)	61.3	(54.7 – 67.6)	28.2	(22.6 – 34.6)
65 – 79 years	53.8	(49.4 – 58.2)	7.3	(5.3 – 9.8)	73.5	(69.4 – 77.1)	19.3	(16.0 – 23.0)
≥ 80 years	50.6	(44.6 – 56.5)	5.3	(3.2 – 8.6)	69.0	(63.3 – 74.2)	25.7	(20.9 – 31.2)
**Education group**								
Low	49.0	(43.8 – 54.2)	7.1	(4.8 – 10.4)	68.2	(63.1 – 73.0)	24.7	(20.3 – 29.6)
Medium	47.5	(42.4 – 52.6)	10.1	(7.3 – 13.8)	65.4	(60.3 – 70.3)	24.5	(20.2 – 29.3)
High	60.4	(54.5 – 66.0)	7.3	(5.0 – 10.5)	71.8	(66.5 – 76.6)	20.9	(16.7 – 25.8)
**Diabetes duration**								
< 5 years	54.8	(47.2 – 62.2)	9.3	(5.8 – 14.5)	64.2	(56.4 – 71.4)	26.5	(20.0 – 34.3)
5 – 14 years	51.2	(45.6 – 56.8)	7.0	(4.6 – 10.6)	69.5	(64.0 – 74.6)	23.4	(18.9 – 28.7)
≥ 15 years	47.2	(42.4 – 52.2)	7.9	(5.6 – 11 .1)	68.8	(64.0 – 73.1)	23.3	(19.4 – 27.7)
**Diabetes-related complications and cardiovascular diseases**								
Yes	35.1	(30.5 – 40.0)	7.8	(5.4 – 11.0)	62.6	(57.6 – 67.4)	29.6	(25.1 – 34.5)
No, but hypertension	61.4	(55.7 – 66.7)	8.0	(5.4 – 11 .8)	71.8	(66.3 – 76.8)	20.1	(15.8 – 25.3)
No, and no hypertension	67.6	(59.6 – 74.7)	9.1	(5.5 – 14.6)	71.2	(63.4 – 78.0)	19.7	(13.9 – 27.1)
**Total**	**50.2**	**(46.9 – 53.5)**	**8.1**	**(6.4 – 10.1)**	**68.0**	**(64.8 – 71.1)**	**23.9**	**(21.1 – 27.0)**

Education group: CASMIN classification [41]; diabetes-related complications: diabetes-related kidney disease, diabetes-related eye disease, diabetes-related nerve disease, diabetic foot or amputation due to diabetes

^*^Number of cases is n < 10; missing values: n = 2 for self-assessed health, n = 7 for self-assessed health compared to the time before the pandemic

**Annex Table 7:** Self-assessed change in health status compared to the time before the COVID-19 pandemic of persons with type 2 diabetes aged 45 years and over, differentiated by SARS-CoV-2 infection, by age group, education group, duration of diabetes and presence of secondary and concomitant diseases (n = 676 women, n = 772 men). Source: GEDA 2021/2022-Diabetes

	Self-assessed general health compared to the time before the COVID-19 pandemic											
	No SARS-CoV-2 infection						SARS-CoV-2 infection					
	Much/slightly better		About the same		Much/slightly worse		Much/slightly better		About the same		Much/slightly worse	
	%	(95 % CI)	%	(95 % CI)	%	(95 % CI)	%	(95 % CI)	%	(95% CI)	%	(95 %-KI)
**Age group**												
45 – 64 years	10.6	(7.1 – 15.7)	61.1	(54.0 – 67.7)	28.3	(22.3 – 35.2)	9.2^*^	(3.1 – 24.1)	62.6	(43.1 – 78.7)	28.3	(14.2 – 48.5)
65 – 79 years	6.8	(4.8 – 9.4)	75.9	(71.8 – 79.6)	17.3	(14.1 – 21.0)	12.2	(5.5 – 24.8)	47.7	(33.2 – 62.5)	40.1	(26.5 – 55.5)
≥ 80 years	5.1	(3.0 – 8.6)	69.0	(63.1 – 74.4)	25.8	(20.8 – 31.6)	7.1^*^	(1.3 – 30.4)	68.4	(45.8 – 84.7)	24.5^*^	(10.6 – 47.1)
**Education group**												
Low	7.1	(4.7 – 10.6)	69.1	(63.7 – 74.0)	23.8	(19.3 – 28.9)	7.5^*^	(2.4 – 21.6)	58.1	(40.3 – 74.0)	34.4	(19.8 – 52.7)
Medium	9.8	(6.9 – 13.8)	67.0	(61.6 – 72.0)	23.2	(18.9 – 28.2)	12.4^*^	(4.9 – 28.1)	52.2	(35.5 – 68.4)	35.4	(21.1 – 52.9)
High	6.8	(4.5 – 10.1)	72.3	(66.7 – 77.2)	21.0	(16.6 – 26.2)	12.1^*^	(4.9 – 26.8)	67.9	(48.0 – 82.9)	20.1^*^	(8.7 – 39.7)
**Diabetes duration**												
< 5 years	8.0	(4.7 – 13.3)	67.0	(58.7 – 74.3)	25.0	(18.3 – 33.2)	21.2^*^	(7.8 – 46.0)	38.8	(18.9 – 63.3)	40.0^*^	(18.7 – 65.9)
5 – 14 years	7.5	(4.9 – 11.4)	69.5	(63.6 – 74.8)	23.0	(18.2 – 28.6)	2.6^*^	(0.6 – 9.6)	68.8	(51.6 – 82.1)	28.6	(15.9 – 46.0)
≥ 15 years	7.6	(5.2 – 11 .0)	69.8	(64.9 – 74.3)	22.6	(18.6 – 27.2)	11 .8^*^	(5.0 – 25.1)	56.8	(39.9 – 72.2)	31.4	(18.2 – 48.6)
**Diabetes-related complications and cardiovascular diseases**												
Yes	7.0	(4.7 – 10.5)	63.5	(58.1 – 68.6)	29.4	(24.7 – 34.7)	14.5	(7.4 – 26.6)	54.0	(38.7 – 68.6)	31.5	(19.4 – 46.7)
No, but hypertension	7.9	(5.2 – 11 .9)	73.6	(67.8 – 78.6)	18.5	(14.1 – 23.9)	9.2^*^	(2.7 – 27.3)	55.9	(36.9 – 73.3)	34.9	(19.1 – 54.9)
No, and no hypertension	9.8	(5.9 – 15.7)	71.2	(63.0 – 78.3)	19.0	(13.0 – 26.8)	1.2^*^	(0.2 – 8.6)	68.2	(40.0 – 87.4)	30.6	(11 .8 – 59.2)
**Total**	**7.9**	**(6.1 – 10.0)**	**69.0**	**(65.6 – 72.2)**	**23.1**	**(20.2 – 26.3)**	**10.1**	**(5.5 – 17.7)**	**57.4**	**(46.5 – 67.7)**	**32.5**	**(23.2 – 43.5)**

Education group: CASMIN classification [41]; diabetes-related complications: diabetes-related kidney disease, diabetes-related eye disease, diabetes-related nerve disease, diabetic foot or amputation due to diabetes

^*^Number of cases is n < 10; missing values: n = 7 for self-assessed health compared to the time before the pandemic, n = 2 for SARS-CoV-2 infection
